# An overview on central nervous system tuberculosis (CNS-TB) focusing on cognitive impairments

**DOI:** 10.1590/1414-431X2025e14570

**Published:** 2026-01-30

**Authors:** G.L. Garcia, B. Regis da Cunha, J.D. Herling, C.M. Gomes, F.C.R. Zucchi

**Affiliations:** 1 Curso de Graduação em Medicina, Faculdade de Ciências da Saúde, Universidade do Estado de Mato Grosso, Cáceres, MT, Brasil; 2 Faculdade de Medicina, Universidade de Brasília, Brasília, DF, Brasil; 3 Centro de Medicina Tropical, Faculdade de Medicina, Universidade de Brasília, Brasília, DF, Brasil

**Keywords:** Tuberculosis, Central nervous system, Meningitis, Neurological disorders, Cognitive impairments

## Abstract

One of the most serious clinical manifestations of tuberculosis (TB) is the central nervous system (CNS) presentation, which results in neurological disorders and cognitive impairments that may lead to reduced social skills. Few studies have assessed TB neuropsychological symptoms after infection. This review article investigated the incidence and spectrum of cognitive impairment related to complications in patients with CNS-TB and compiled data on the pathophysiology, diagnosis, and treatment of the disease. An extensive literature review was performed, and a total of 286 published studies were selected for manual screening. For analysis purposes, 43 studies were included in this review. CNS-TB mainly affects young children and is fatal in over 50% of cases, with survivors showing high morbidity. The characteristics of this disease include meningitis and brain tissue granulomas. This leads to extensive neurological involvement, resulting in a complex mechanism that alters the structure and composition of cells in the brain including the cerebellum and spinal cord. It also impairs language development, reading, and learning complex tasks, and therefore affects the patient's social adjustment. The results of our review provide information connecting the basis of neuroscience and clinical medicine, especially childcare. Furthermore, early diagnosis is imperative to prevent serious cognitive consequences of TB in the developing CNS.

## Introduction

Tuberculosis (TB) is an ancient human disease caused by *Mycobacterium tuberculosis* (or Koch's *bacillus*), which primarily affects the lungs, making lung disease the most common presentation (1). However, central nervous system tuberculosis (CNS-TB), particularly meningeal involvement, is the most severe form of extrapulmonary TB, due to the high degree of morbidity and mortality (2).

A quarter of the world's population is infected with *M. tuberculosis* and 5-10% of these individuals develop TB disease during their lifetime. CNS-TB occurs in 1% of all cases and is the most devastating form of TB, particularly tuberculous meningitis (MTB). Given the 10 million new TB patients annually, approximately 100,000 new CNS-TB cases were estimated in 2019 (2,3).

In surviving patients, neurological sequelae are diverse, including motor and sensory disorders, hydrocephalus, and cognitive impairment (4,5), which lead to a gradual and progressive loss of mental functions in the domains of memory, orientation, language, reasoning, executive functions, learning, and visuospatial skills (6). Although sequelae seem to be multicausal, systemic and central nervous system infections, including human immunodeficiency virus (HIV) infection (7), seem to play an important role.

For this study, the available information on various aspects of cognitive impairment in CNS-TB was collected. An extensive literature review of articles published in English, Portuguese, and Spanish was carried out in the Scopus, LILACS, and PubMed databases. Search terms included “tuberculosis of the central nervous system”, “neurotuberculosis”, and “tuberculous meningitis”. A total of 286 published articles/reports were selected for screening. For analysis purposes, this review included 43 publications for their relevance and presenting complete information on cognitive impairment in CNS-TB as well as the epidemiology, pathophysiology, diagnosis, and treatment of this disease.

## Epidemiology

TB is still one of the ten leading causes of death in the world (8) and the second most common cause of death due to a single infectious agent after COVID-19 (9). Every year, 10 million people fall ill and, despite being a preventable and curable disease, 1.5 million people die each year from tuberculosis (8).

Although TB is prevalent throughout the world, developing countries are responsible for a disproportionate share of the TB burden (1). Furthermore, children under 5 years of age and immunosuppressed patients have a considerably higher risk of CNS-TB (3,10-13). Among them, HIV infection is a known risk factor for the development of extrapulmonary TB, including CNS-TB. Therefore, in areas with low HIV control, the incidence of CNS-TB is still high (2,13). The use of immunosuppressive agents, such as long-term corticosteroid therapy, has also been considered a risk factor (1).

## Pathogenesis and pathology

*M. tuberculosis* is an acid- and alcohol-resistant intracellular bacillus. The bacillus has several unique characteristics compared to other bacteria, including the high lipid content of its cell wall, which contributes antibiotic resistance, hampers Gram staining, and enhances its ability to survive in extreme conditions (1).

TB infection occurs by inhalation of droplets infected with *M. tuberculosis*, which cross the lung epithelium and infect alveolar macrophages, neutrophils, and dendritic cells (6). Several mechanisms by which bacilli migrate to the lymphatic system or bloodstream have been suggested: by invading and crossing vascular endothelial cells, by replicating in lymphatic endothelial cells, and by being transported to distant sites in phagocytes (6,14,15).

Once the bacilli gain access to the brain, they survive the limited local innate immunity, replicate, and develop into silent, encapsulated tuberculous lesions (latent TB) (16). Rich and McCordock (17) suggested that CNS-TB starts with the rupture of one of these lesions, called the Rich focus, located under the cortical pia or adjacent to the meninges or ventricles, releasing TB bacilli into the subarachnoid space, causing a granulomatous infection of the meninges.

The interaction between the *bacillus* and the host immune response (active TB) can be fatal: once these granulomatous foci rupture (possibly due to a decrease in host immunity) and reach the brain or meninges (16), they are recognized by microglia, which initiate a harmful immune-inflammatory response, including secretion of inflammatory cytokines, proteases, lipid mediators, neuromarkers, and tryptophan metabolites, triggering MTB (13,17-20). Granulomatous foci (Rich foci) form around the bacilli to contain the infection. Rupture of these foci into the subarachnoid space triggers a severe inflammatory response, leading to the development of CNS-TB (14).

Although cytokines play a critical role in host defense against TB infection, they can also mediate inflammation. Tumor necrosis factor is central to the pathogenesis of TB-CNS: it increases the permeability of vessels allowing immune mediators to cross the blood-brain barrier (BBB) and reach the CNS (6,14,21,22).

The characteristics of CNS-TB, in addition to the meningeal inflammation, include vasculitis, hydrocephalus, and basal exudate (6,10,14,16,23). The exudate is thick and gelatinous, composed of fibrin, lymphocytes, plasma cells and other mononuclear cells, polymorphonuclear leukocytes, and some areas of caseous necrosis. The exudate surrounds the optic chiasm, the base of temporal structures in the subarachnoid space, and the base of the skull, spreading around the medulla oblongata, floor of the third ventricle, and subthalamic region, which can lead to blockage of cerebrospinal fluid (24).

Vascular endothelial growth factor plays diverse roles in vasculogenesis and angiogenesis, decreases BBB permeability, and is considered a useful biomarker of TB, indicating disease activity vs latent disease (6,21).

Finally, an observational cohort study demonstrated a significant role of tryptophan metabolism as an indicator of CNS-TB outcome: its concentration in the cerebrospinal fluid (CSF) was lower in individuals who survived than in those who died. More than 10 gene loci have been identified as being predictive of CSF tryptophan concentrations, suggesting the influence of host genotype on CNS-TB outcome (13).

## Clinical findings

CNS-TB has three recognizable phases. The illness begins with malaise, fatigue, low-grade fever, and intermittent headache, sometimes associated with vague neck or back discomfort and mild personality change (16,23). Within 2 to 3 weeks, the patient experiences prolonged headache, meningismus, vomiting, subtle confusion, and varying degrees of paralysis. Thereafter, the disease can rapidly accelerate to the paralytic phase: delirium followed by stupor and coma, seizures, multiple cranial nerve deficits, hemiparesis, and hemiplegia. In untreated cases, death commonly occurs within 5 to 8 weeks of illness onset. In children, headache is less common, while irritability, restlessness, anorexia, and prolonged vomiting are prominent symptoms (16).

Careful attention to the past, the epidemiological setting, and the general physical examination can reveal essential information. A strong family history of TB is helpful in the diagnosis. Recent exposure to people with active TB is also a risk factor in cases involving children and in adults with impaired cellular immunity. In urban areas, the association of extrapulmonary TB with underlying conditions such as alcoholism, injecting drug use, poverty, and other conditions or therapies that impair cellular immune function help diagnose the condition (3,16,23). Signs of active infection elsewhere in the body are frequent and, when present, provide the most reliable basis for presumptive diagnosis in patients who present with meningeal TB (16).

The key to diagnosis in most cases lies in the proper interpretation of CSF cellular and biochemical characteristics combined with testing for CSF mycobacteria by the acid-fast stain test (16). Another useful diagnostic tool is the measurement of adenosine deaminase in the CSF, but the result must be interpreted with caution, as its elevated level also occurs in other bacterial infections, without a clear threshold to distinguish MTB from a different bacterial meningitis (3).

CNS-TB diagnosis based on CSF examination includes a differential cell count and the analysis of protein and glucose levels. CNS-TB presents leukocytosis with a predominance of lymphocytes, elevated levels of protein, plasma glucose below 50%, and mononuclear pleocytosis or a transient polymorphonuclear predominance (3,16,23).

The clinical and biological picture is commonly misinterpreted as bacterial meningoencephalitis, leading to clinical worsening and delaying diagnosis and treatment. The differential diagnosis is broad and includes other causes of basilar meningitis, including sarcoidosis, neurosyphilis, fungal meningitis, viral meningitis, autoimmune encephalitis, neurobrucellosis, and leptomeningeal carcinomatosis (3).

The Ziehl-Neelsen CSF staining technique for acid-fast bacilli (CBAR) is inexpensive and widely available, but it has a low sensitivity unless performed by experienced microscopists using large volumes of CSF centrifuged at high speeds to concentrate *M. tuberculosis* (13). Ink staining is used to exclude cryptococcosis (25) and the diagnosis, according to some authors, is best made with a lumbar puncture (16,23). Additional tests include the Mantoux reaction (tuberculin skin test; Purified Protein Derivative, PPD) and bacteriological sputum analysis (26,27), which may support the diagnosis of tuberculosis although they do not confirm CNS-TB.

In summary, testing CBAR in CSF remains the fastest means of reaching an early diagnosis (1,27). The sensitivity of the CBAR and culture can vary, influenced in part by the selection and volume of the specimen provided by the clinician, as well as the attention to detail of the laboratory staff in processing the samples. Submitting multiple CSF samples from repeated lumbar punctures can be helpful. There is no need to postpone anti-TB therapy, as the diagnostic yield remains good for several days after treatment initiation (16).

Polymerase chain reaction (PCR) is an effective method for the rapid detection of *M. tuberculosis* DNA in CSF (1,27). However, its reliability is not well established, mainly due to variable sensitivity and specificity among laboratories (16). The detection of protein B antigen (PAB) by PCR has proven to be an effective and rapid method for diagnosing CNS-TB. It demonstrated higher sensitivity (82%) and negative predictive value (81%) compared to other tests, including PCR IS6110. Therefore, PAB PCR can be used as a simple, cost-effective, and rapid diagnostic tool to enhance the clinician's ability to detect CNS-TB (28). The World Health Organization currently recommends the Xpert MTB/RIF (*M. tuberculosis*/rifampin) assay as the initial molecular diagnostic test for CSF specimens from patients with suspected MTB. It is an automated cartridge-based PCR that simultaneously detects resistance to *M. tuberculosis* and RIF in less than two hours (1,27,29).

The application of computed tomography (CT) and magnetic resonance imaging (MRI) has significantly improved the assessment and management of patients with CNS-TB (27). These imaging studies are useful for identifying the presence and extent of basal arachnoiditis, cerebral edema, and infarction, and the presence/level of hydrocephalus. In a patient with the characteristic clinical features, a CT showing any degree of hydrocephalus is strongly suggestive of MTB. If the CT is normal at the time therapy is started, the prognosis for complete recovery from therapy is excellent. MRI is superior to CT for evaluating children and is the preferred modality for evaluating brainstem, midbrain, and basal ganglia lesions in patients of all ages (16).

## Central nervous system disorders

CNS-TB can have three clinical forms: MTB, intracranial tuberculoma, and spinal arachnoiditis. MTB is more prevalent in the western world and presents as a syndrome with meningitis that can vary from subacute to chronic, a prodrome of malaise, fever and headache progressing to altered consciousness and focal neurological signs, followed by stupor, coma, and death within five to eight weeks of onset (3,16).

Most therapeutic advances have been derived from studies in adult patients; however, pharmacokinetics and treatment outcomes are different in children. Children generally metabolize drugs differently, and the rate of drug release is inversely proportional to age (13,30).

The clinical stage of the patient at the initiation of therapy has a significant impact on both mortality and the incidence of subsequent neurological sequelae. These include cranial nerve palsies, gait disturbances, hemiplegia, blindness, deafness, learning disabilities, dementia, and various syndromes of hypothalamic and pituitary dysfunction (16). In a case report, a 22-year-old male patient showed cognitive disorders such as apathy, psychomotor retardation, and impaired memory (31).

Lin et al. (32) suggest that abnormalities in the limbic system and globus pallidus may be a specific biomarker for cognitive function in bipolar disorder. Similarly, the limbic system has been studied in patients with CNS-TB. The authors explain that CNS-TB and cryptococcal meningitis are the two most common types of chronic meningitis. A study of 19 patients with chronic CNS-TB showed a decline in white matter microstructural integrity in several limbic regions including the cingulate gyrus and parahippocampal gyrus, which was associated with worse CSF analysis profiles.

Chen et al. (5) support that patients with CNS-TB had worse performance in neuropsychological tests and lower gray matter volume in the right thalamus, right caudate nucleus, right superior and middle temporal gyrus, right precuneus, and left putamen compared to healthy controls.

Multiple impairments of cognitive function may persist in patients with chronic CNS-TB, even after adequate treatment. Severe early illness may contribute to increased vulnerability to brain tissue damage, with subsequent neuropsychological consequences (5).

In the spinal cord, CNS-TB produces inflammatory exudates and complications such as tuberculous radiculomyelitis (the most common complication, occurring in 38.7% of patients), spinal cord infarcts, tuberculous myelitis, intramedullary medullary tuberculoma, syringomyelia (late complication seen in approximately 15% of patients), and spinal tuberculous abscess (in 7.2% of patients). Vertebral body infection or Pott's disease is the most common cause of myelopathy in patients with CNS-TB, and tuberculous arachnoiditis leading to myeloradiculopathy is the most characteristic spinal complication (33).

In MTB, patients with hydrocephalus, cerebral infarction, paradoxical reactions including tuberculomas, neurological immune reconstitution inflammatory syndrome in HIV-coinfected individuals, and seizures have a lower Glasgow Coma Scale score, a tool routinely used for patient assessment. The cause of these findings in MTB are multifactorial and data regarding the cause and timing of seizures in patients are scarce (13).

Tuberculoma, a rare condition found in 1% of CNS-TB, usually occurs in immunocompromised patients, such as HIV patients (34). Focal brain injury involves the process of hematogenous spread from a primary focus to other organs, such as the lung. With low cell-mediated immunity and significant inoculation size, tuberculosis foci in the brain parenchyma can develop into a tuberculoma or abscess (16,34,35).

The usual clinical picture is a child or young adult presenting with headache, seizures, progressive hemiplegia, and/or signs of elevated intracranial pressure, with no symptoms of systemic infection or signs of meningitis in most cases (33,34).

Finally, tuberculous spinal arachnoiditis is a late complication of CNS-TB. The resulting inflammatory reaction is usually local and progresses gradually over weeks to months, producing partial or complete encasement of the spinal cord by a gelatinous or fibrous mass (16). Patients usually show some combination of nerve root involvement and signs of spinal cord compression with the advancement of arachnoiditis. Symptoms include pain, hyperesthesia or paresthesia along the nerve root, lower motor neuron palsy, and bladder sphincter or rectal sphincter incontinence. Localized vasculitis can result in anterior spinal artery thrombosis and cord infarction (16).

A diagnosis of spinal TB arachnoiditis should be considered for a patient with any combination of the following clinical and laboratory features: subacute onset of spinal or nerve root pain, rapid ascending transverse myelopathy or multilevel myelopathy, increased CSF protein concentration and cell count, MRI signs of arachnoiditis or epidural space infection, and evidence of TB elsewhere in the body. Tissue biopsy for histopathology and culture stains is required for diagnosis (16).

Spinal TB arachnoiditis can progress to various clinical conditions such as radiculomyelitis, spinal tuberculoma, myelitis, syringomyelia, vertebral tuberculosis, or spinal tuberculosis abscess. Therefore, prompt treatment with a first line antituberculosis drug (isoniazid, rifampicin, ethambutol, pyrazinamide, and streptomycin) is important (34).

Several studies have shown that cognitive deterioration is common in people with HIV, especially in people who are not yet on treatment (36): effective antiretroviral treatment greatly delays cognitive impairment (37). Cognitive impairment was slightly greater in patients with HIV/AIDS (51.3%) than in patients without HIV co-infection (47.7%); however, in patients with HIV/AIDS, moderate damage predominated (55%) and, in patients without HIV, severe impairment was more common (58.1%). Cognitive sequelae occurred in both groups, without differences.

Although CNS-TB in patients with HIV/AIDS is associated with a higher risk of cognitive impairment, a greater degree of severity or sequelae has not been proven in these patients. During follow-up, an improvement in previously defined sequelae was observed in both groups (with and without HIV infection), with higher scores in patients with HIV/AIDS (38).

## Therapy

Current treatments of MTB can generally be separated into those that focus on optimizing the erradication of *M. tuberculosis* and those that use adjuvant therapies to prevent and manage disease complications (13).

As emphasized, the first principle of TB therapy is that it should be started based on strong clinical suspicion rather than postponed until the final diagnosis is obtained. The prognosis is good when treatment is started before the development of focal neurological signs and changes in consciousness levels. No randomized trials have yet established the optimal combination of drugs, dosing, and treatment duration for MTB (3,16).

The principles governing therapy for MTB are those derived from the management of pulmonary TB: combined therapy is always indicated, and monotherapy should never be used (1). The aim of using drug combination regimens is to enhance the bactericidal effect, prevent drug resistance, and reduce the likelihood of therapy-resistant pathogens (3,16).

A combination of first- and second-line drugs is often used to treat this condition (1,23,27). First-line drugs include isoniazid, rifampicin, rifabutin, rifapentine, pyrazinamide, and ethambutol. Second-line TB drugs include injectable aminoglycosides such as amikacin, kanamycin, and streptomycin, as well as injectable polypeptides such as capreomycin and viomycin (1).

Given rifampicin's poor CNS penetration and its importance as a first-line therapy, several studies with this high-dose drug, with or without linezolid, are ongoing. High-dose isoniazid therapy in rapid acetylators is also under investigation (13). It is worth noting that liver function testing is required for all patients taking isoniazid and monitoring for retinopathies is important for patients taking ethambutol (1).

In 2012, the US Food and Drug Administration approved bedaquiline as a drug for the treatment of multidrug-resistant TB (1). Despite advances in TB chemotherapy, mortality from MTB remains high (adults 50%, children 20%) (39,40). Mortality in children is lower than in adults, but because their brain is developing, children are at risk of developing age-specific neurological sequelae (3,13,16).

Adjuvant corticosteroid therapy is widely used in the treatment of CNS-TB, as it reduces inflammation, limits CNS damage, and thus improves outcome and reduces MTB mortality, at least in the short term (13,16,41). This therapy is recommended for all patients with significant clinical evidence of CNS-TB, the possible exception being adults with mild stage 1 disease. Corticosteroids appear to be more beneficial in cases with some complications, such as increased intracranial pressure, cerebral edema, and spinal block (16,42).

An interesting fact about the pathology of CNS-TB is that intracranial tuberculomas seem, paradoxically, to increase in size as patients are treated, a phenomenon known as paradoxical expansion (14,27,35). This could be due to immune reconstitution or resistance to antimycobacterial drugs of TB bacilli. Once active disease is treated, immune reconstitution occurs with return of delayed-type hypersensitivity to TB bacilli and antigens, leading to macrophage and lymphocyte activation and the formation of granulomas and tuberculomas. Therefore, intensifying TB therapy during a paradoxical response may not be logical (14).

Ventriculoperitoneal shunt, the usual treatment for hydrocephalus, should not be the first line of treatment for hydrocephalus in MTB. Catheter obstruction and secondary infections are common in MTB patients and lead to high morbidity and mortality. In a study of 65 patients, the complication rate was up to 32.3%. Therefore, this therapy should be reserved for cases of therapy resistance and, obviously, for emergency cases of acute obstructive hydrocephalus (14).

Adjunctive aspirin therapy in MTB has been shown to decrease the incidence and promote resolution of cerebral infarcts (13,39).

Finally, bacillus Calmette-Guérin (BCG) is effective in protecting infants and children against severe miliary and meningeal TB, and children with CNS-TB who were immunized seem to maintain better mental activity and have better outcomes (21,43). Although the protective efficacy of BCG is variable in adults, it seems to prevent the spread of TB, including miliary TB (32).

## Conclusions

Tuberculosis is a preventable and treatable infectious disease, yet it is still a major contributor to morbidity and mortality in developing countries, where we are still struggling to provide adequate access to care. Some of the challenges are lack of awareness, delay in diagnosis, and poor medication adherence.

Prevention is important to reduce disease case numbers and early diagnosis helps to reduce the cognitive impairment caused by CNS-TB. However, the differential diagnosis is difficult, since CNS-TB can mimic other conditions such as viral meningitis or even the common cold.

Future research may indicate a universal criterion regarding the stage of recovery after brain injury, which is still unknown. The patients may present sequelae, which indicates a partial recovery, which can sometimes go unnoticed in the outpatient clinic. One way to assess the patient's condition is by assessing if they are able to resume the activities they engaged in before the illness.

Finally, this review shows the importance of more investment in the development of strategies and research to eradicate this disease from the world map. New antituberculosis drugs need to be developed to shorten and simplify the treatment of TB caused by drug-susceptible organisms, improve the treatment of drug-resistant TB, and provide more efficient and effective treatment of latent TB infection.

## Data Availability

All data generated or analyzed during this study are included in this published article.
